# Supplementary material for: Gut Dysbiosis and Neurobehavioral Alterations in Rats Exposed to Silver Nanoparticles
Source: Sci Rep. 2017 Jun 6;7:2822. doi: 10.1038/s41598-017-02880-0 (PMC5460200; doi:10.1038/s41598-017-02880-0)
Supplement: Supplementary file 1 — Supplementary Material [file 41598_2017_2880_MOESM1_ESM.doc]

**Supplementary Information**

**Gut Dysbiosis and Neurobehavioral Alterations in Rats**

**Exposed to Silver Nanoparticles**

Angela B. Javurek^1,#^, Dhananjay Suresh^2,#^, William G. Spollen^3,4^, Marcia L. Hart^5^,

Sarah A. Hansen^6^, Mark R. Ellersieck^7^, Nathan J. Bivens^8^, Scott A. Givan^3,4,9^,

Anandhi Upendran^10,11,*^, Raghuraman Kannan^11,12*^, Cheryl S. Rosenfeld^4,13,14,15 *^

**Departments of:**

^1^ Occupational and Environmental Health Sciences, West Virginia University, Morgantown, WV 26506 USA

^2^ Biological Engineering, University of Missouri, Columbia, MO 65211 USA

^3^ Informatics Research Core Facility, University of Missouri, Columbia, MO 65211 USA

^4^ Bond Life Sciences Center, University of Missouri, Columbia, MO 65211 USA

^5^ Veterinary Pathobiology, University of Missouri, Columbia, MO 65211 USA

^6^ Office of Animal Resources, University of Missouri, Columbia, MO 65211 USA

^7^ Agriculture Experimental Station-Statistics, University of Missouri, Columbia, MO 65211 USA

^8^ DNA Core Facility, University of Missouri, Columbia, MO 65211 USA

^9^ Molecular Microbiology and Immunology, University of Missouri, Columbia, MO 65211 USA

^10^ Medical Research Office, University of Missouri, Columbia, MO 65211 USA

^11^ Department of Medical Pharmacology and Physiology, University of Missouri, Columbia, MO 65211 USA

^12^ Radiology, University of Missouri, Columbia, MO 65211 USA

^13^ Biomedical Sciences, University of Missouri, Columbia, MO 65211 USA

^14^ Genetics Area Program, University of Missouri, Columbia, MO 65211 USA

^15^ Thompson Center for Autism and Neurobehavioral Disorders, University of Missouri, Columbia, MO 65211, USA

^#^These authors contributed equally to the manuscript.

**Short Title:** Silver nanoparticles and the gut-microbiome-brain axis

**Keywords:** Environmental chemicals, rodents, antimicrobials, bioinformatics, anxiety, microbiota, bacteria, elevated plus maze, and histopathology

***Co-corresponding authors**: [upendrana@health.missouri.edu](mailto:upendrana@health.missouri.edu), [KannanR@health.missouri.edu](mailto:KannanR@health.missouri.edu), and [rosenfeldc@missouri.edu](mailto:rosenfeldc@missouri.edu).

**Supplementary Figures**


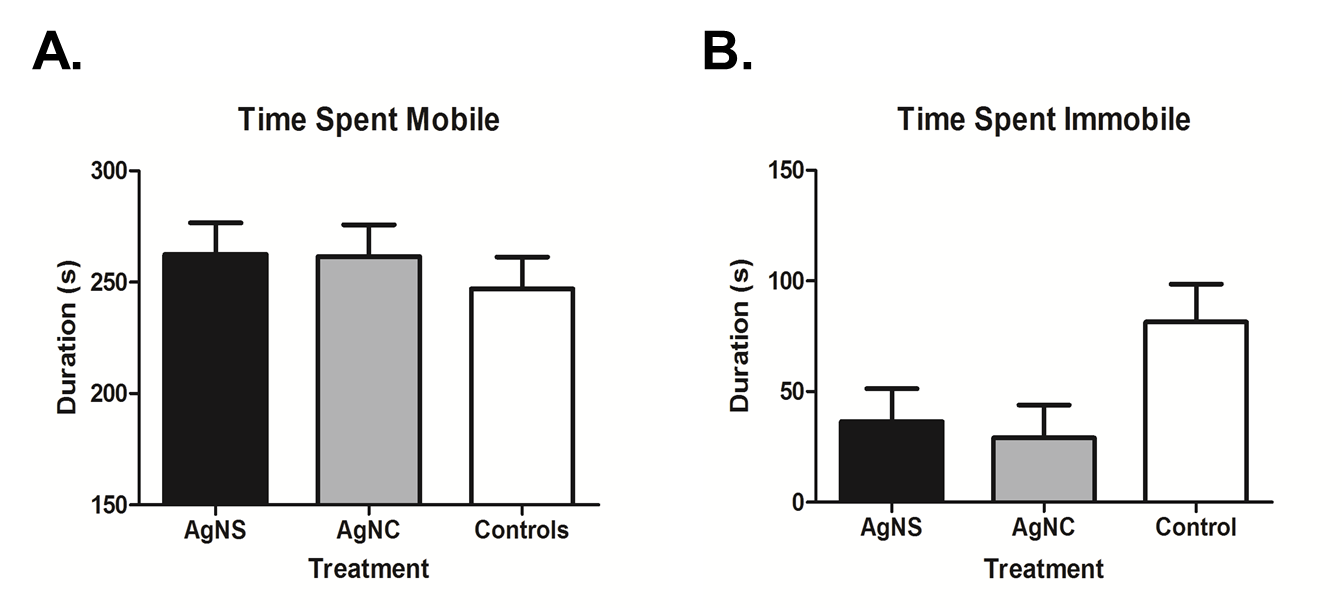
**Supplementary Figure 1.** Time spent mobile and immobile in the EPM. A) Time spent mobile. B) Time spent immobile. No differences based on treatment were observed for either of these behaviors.


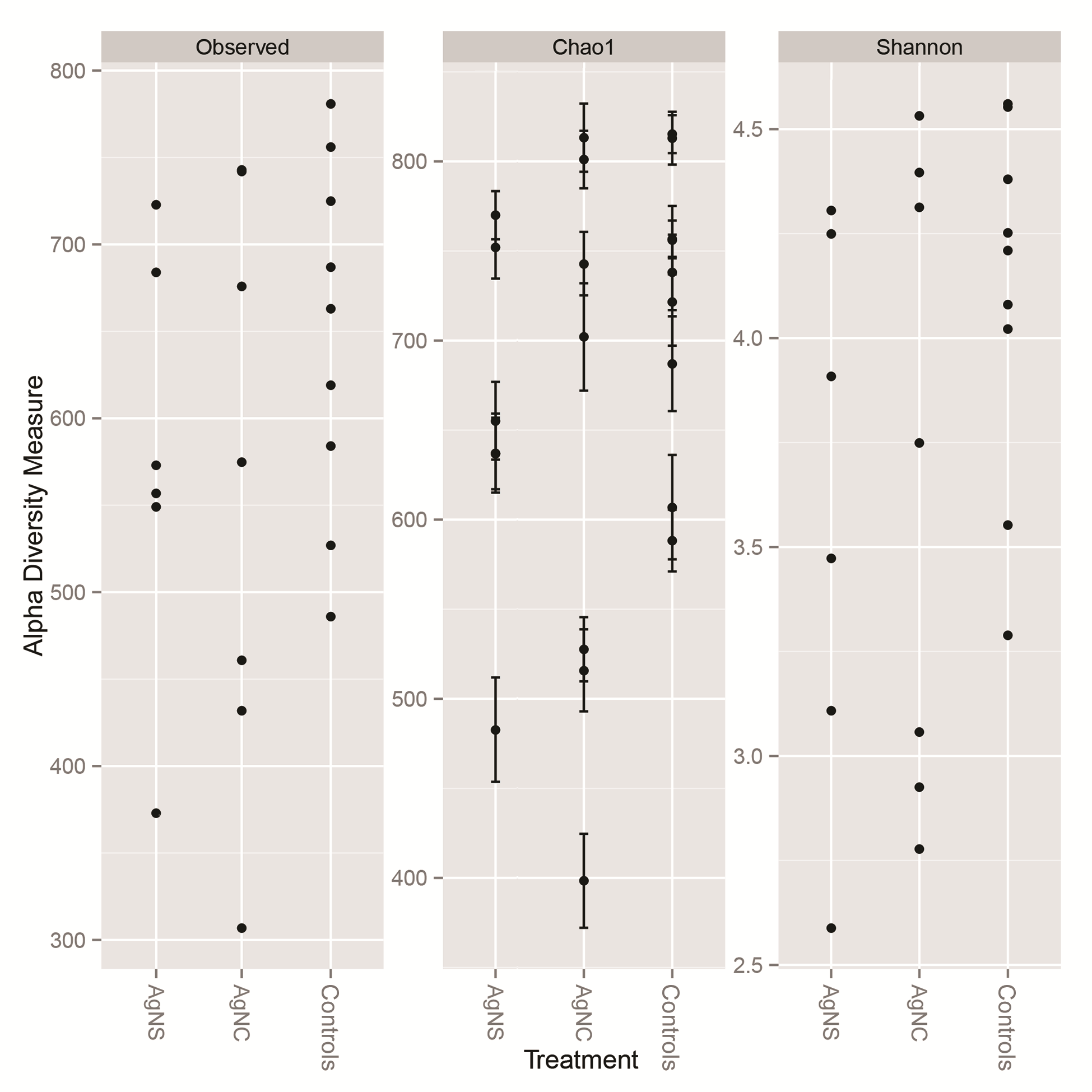
**Supplementary Figure 2.** Alpha-diversity analyses of fecal samples from AgNC, AgNS, and control groups. Total observed species and species richness (Chao1) and diversity (Shannon) indices for all three groups plotted using the phyloSeq R package plot_richness function ^1^. In each graph, the mean is represented by a circle, and the error bars represent the SEM for the Chao1 data, as shown in the middle graph. The observed value detailed in the first graph is simply the total species count for each sample and is a single measure. The Chao1 index estimates the species richness by adding a correction factor to the observed number of species. This correction factor is a function of the number of species that occur just once and those that occur twice in a sample. These two measures are also used to estimate a variance for each sample, which gives rise to the depicted error bars. Shannon measures the evenness of each sample. It is based on the relative abundances of the OTUs in each sample, and thus, it does not include an estimate of variance.

**
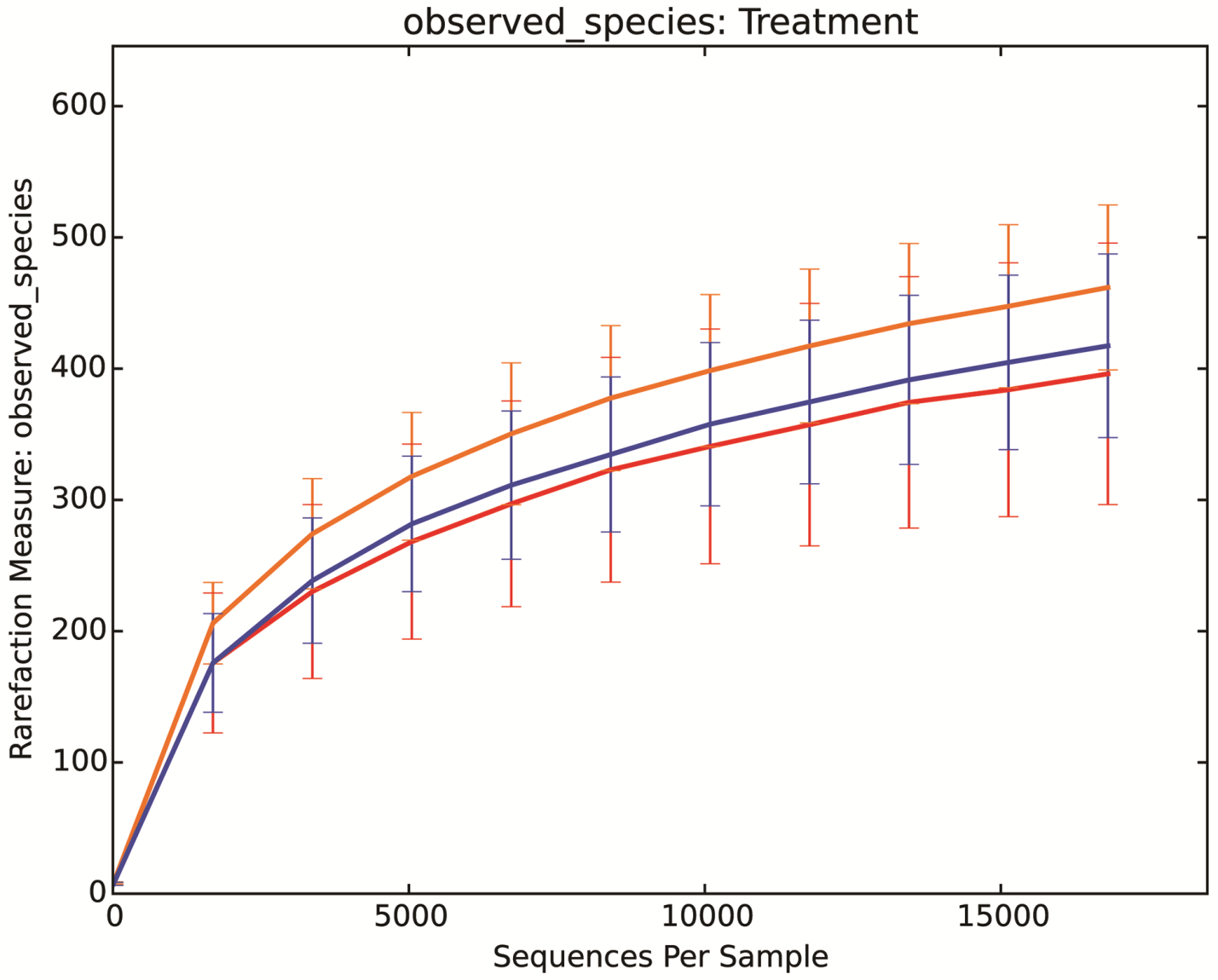
**

**Supplementary Figure 3.** Rarefaction metrics plotted for aggregate fecal samples using the alpha_rarefaction.py script in the Qiime package ^2^.


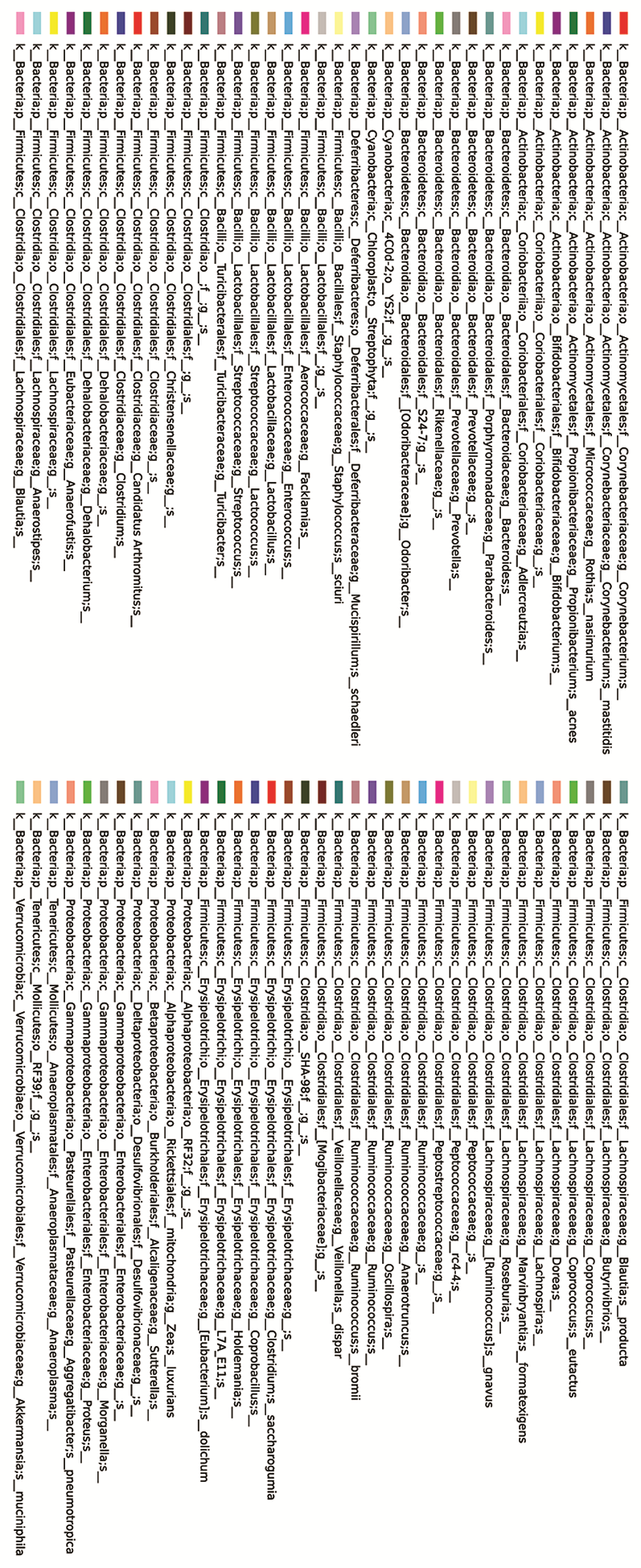
**Supplementary Figure 4.** Key for the bacterial species listed in Fig. 2.


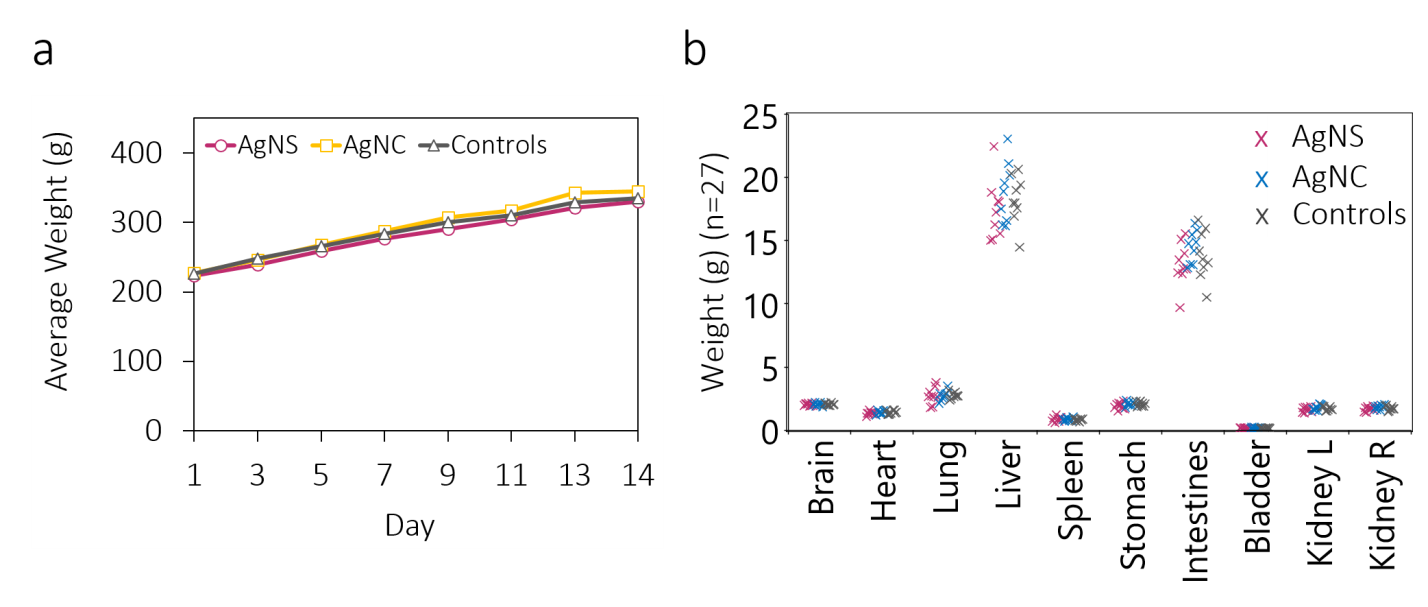
**Supplementary Figure 5.** Average body and organ weights for AgNP, AgNC, and vehicle control (water) rat groups. A) Average body weights. B) Average organ weights. No differences based on treatment were detected for average body weight or organ weights.

**References Cited in Supplementary Information**

1 McMurdie, P. J. & Holmes, S. phyloseq: an R package for reproducible interactive analysis and graphics of microbiome census data. *PLoS One* **8**, e61217, doi:10.1371/journal.pone.0061217 (2013).

2 Caporaso, J. G. *et al.* QIIME allows analysis of high-throughput community sequencing data. *Nat Methods* **7**, 335-336, doi:10.1038/nmeth.f.303 (2010).
